# Supplementary material for: Identification of photoperiod-induced specific miRNAs in the adrenal glands of Sunite sheep (Ovis aries)
Source: Front Vet Sci. 2022 Jul 22;9:888207. doi: 10.3389/fvets.2022.888207 (PMC9354845; doi:10.3389/fvets.2022.888207)
Supplement: Supplementary file 1 [file Data_Sheet_1.ZIP › Supplementary file/miRDeep2 file.zip!/miRDeep2 file/Know_Structure/SPLP42A2/oar-mir-432.pdf]

miRBase precursor	: oar-mir-432
Total read count	: 2218
oar-miR-432 read count	: 2209
remaining reads	: 9

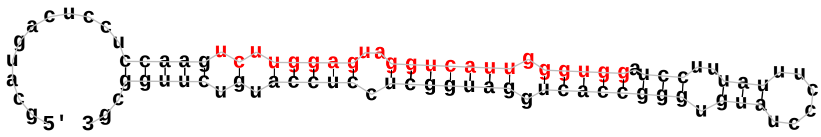

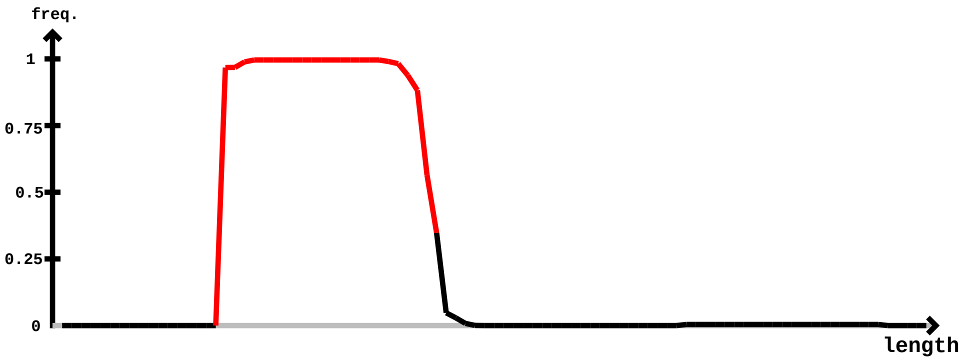


```
                                oar-miR-432
5'- gcaugacuccuccaagucuuggaguaggucauuggguggauccuuuauuucccuaugugggccacuggauggcuccuccaugucuuggcg   -3'    exp
    ...........(((((.(.(((((..(((((((.(((((.(((..(((......))).)))))))).))))))).))))).).)))))..    reads      mm         sample
    ................ucuuggaguaggucauu.........................................................    13         0          seq
    ................ucuuggaguaggucauug........................................................    18         0          seq
    ................ucuuggaguaggucauugg.......................................................    97         0          seq
    ................ucuuggaguaggucauuggg......................................................    124        0          seq
    ................ucuuggaguaggucauugggu.....................................................    704        0          seq
    ................ucuuggaguaggucauugggug....................................................    480        0          seq
    ................ucuuggaguaggucauuggguggg...................................................    657        0          seq
    ................ucuuggaguaggucauuggguggа..................................................    36         0          seq
    ................ucuuggaguaggucauuggguggau.................................................    17         0          seq
    .................cuuggaguaggucauugggugg...................................................    1          0          seq
    ..................uuggaguaggucauugggug....................................................    1          0          seq
    ..................uuggaguaggucauugggugg...................................................    6          0          seq
    ..................uuggaguaggucauuggguggа..................................................    5          0          seq
    ..................uuggaguaggucauuggguggau.................................................    24         0          seq
    ..................uuggaguaggucauuggguggauc................................................    9          0          seq
    ..................uuggaguaggucauuggguggaucc...............................................    1          0          seq
    ...................uggaguaggucauugggug....................................................    1          0          seq
    ...................uggaguaggucauugggugg...................................................    1          0          seq
    ...................uggaguaggucauuggguggа..................................................    1          0          seq
    ...................uggaguaggucauuggguggau.................................................    5          0          seq
    ...................uggaguaggucauuggguggauc................................................    8          0          seq
    ................................................................cuggauggcuccuccaugucu.....    9          0          seq
```
